# Antioxidant-Conjugated Peptide Attenuated Metabolic Reprogramming in Pulmonary Hypertension

**DOI:** 10.3390/antiox9020104

**Published:** 2020-01-25

**Authors:** Mathews Valuparampil Varghese, Maki Niihori, Cody A Eccles, Sergey Kurdyukov, Joel James, Olga Rafikova, Ruslan Rafikov

**Affiliations:** Department of Medicine, Division of Endocrinology, University of Arizona College of Medicine, Tucson, AZ 85721, USA; mathewsv@email.arizona.edu (M.V.V.); mniihori@email.arizona.edu (M.N.); codyeccles@email.arizona.edu (C.A.E.); sergey.kurdyukov@sydney.edu.au (S.K.); joeljames@deptofmed.arizona.edu (J.J.)

**Keywords:** Pulmonary arterial hypertension, metabolism, Warburg effect, anaplerosis, nitroxide-conjugated peptide, Akt

## Abstract

Pulmonary arterial hypertension (PAH) is a chronic cardiopulmonary disorder instigated by pulmonary vascular cell proliferation. Activation of Akt was previously reported to promote vascular remodeling. Also, the irreversible nitration of Y350 residue in Akt results in its activation. NitroAkt was increased in PAH patients and the SU5416/Hypoxia (SU/Hx) PAH model. This study investigated whether the prevention of Akt nitration in PAH by Akt targeted nitroxide-conjugated peptide (NP) could reverse vascular remodeling and metabolic reprogramming. Treatment of the SU/Hx model with NP significantly decreased nitration of Akt in lungs, attenuated right ventricle (RV) hypertrophy, and reduced RV systolic pressure. In the PAH model, Akt-nitration induces glycolysis by activation of the glucose transporter Glut4 and lactate dehydrogenase-A (LDHA). Decreased G6PD and increased GSK3β in SU/Hx additionally shunted intracellular glucose via glycolysis. The increased glycolytic rate upregulated anaplerosis due to activation of pyruvate carboxylase in a nitroAkt-dependent manner. NP treatment resolved glycolytic switch and activated collateral pentose phosphate and glycogenesis pathways. Prevention of Akt-nitration significantly controlled pyruvate in oxidative phosphorylation by decreasing lactate and increasing pyruvate dehydrogenases activities. Histopathological studies showed significantly reduced pulmonary vascular proliferation. Based on our current observation, preventing Akt-nitration by using an Akt-targeted nitroxide-conjugated peptide could be a useful treatment option for controlling vascular proliferation in PAH.

## 1. Introduction

Pulmonary arterial hypertension (PAH) is a rare disease that rapidly progresses by increased proliferation in pulmonary vascular cells causing irreversible vascular remodeling. This accounts for increased pulmonary arterial pressure and later cardiac dysfunction, the leading cause of patient mortality in PAH [1]. According to Hoeper et al. [2], the frequency of PAH is 1.1 to 17.6 per million adults per year and has an occurrence of 6.6 to 26 individuals per million. Even though the disease remains incurable, despite the ineffective existing therapy, the only alternative treatment for this disease is a heart and lung transplantation. Unfortunately, this option is only accessible in designated surgical centers, and only half of these patients survive the surgery with a lifespan of approximately five years [3,4].

Akt signaling plays an important role in the pathogenesis of hypoxia-induced PAH [5]. In PAH, Akt activation results in the stimulation of cell survival by preventing apoptotic pathways and activating prosurvival mechanisms. Recently, we identified that the nitration of Akt1 at Tyr350 causes its activation, ensuing greater endothelial nitric oxide synthase (eNOS) phosphorylation, subsequent mitochondrial translocation, and affecting mitochondrial function [6]. Increased nitric oxide (NO) production augmented high levels of peroxynitrite formation, nitrosative stress, and enhanced protein nitration, which was reported in PAH [7]. These altered proteins may affect many important cytosolic and mitochondrial metabolic pathways; therefore, controlling nitrosative stress is an effective option in preventing PAH progression.

Mitochondria are an important source of reactive oxygen species (ROS), which accounts for vascular dysfunction in PAH [8]. Under conditions of chronic hypoxia, hypoxia-inducible factor and eNOS regulate various cellular functions to meet increased cellular bioenergetic requirements in the pathogenesis of PAH [9,10]. Mitochondrial dysfunction limits ATP production, which results in a metabolic shift towards the Warburg effect often found in highly proliferative cancer cells and pulmonary vascular cells [11]. Another factor inducing cellular proliferation is anaplerosis, which is the replenishment of tricarboxylic acid (TCA) cycle carbon intermediates via the glutaminase-mediated deamidation of glutamine or the carboxylation of pyruvate [12]. Recent studies have linked anaplerosis and glutaminolysis (anabolic pathways that promote cellular biomass for highly proliferative tumor cells) to the hyperproliferative state of PAH, leading to hypertrophy and pulmonary vascular resistance [13].

Antioxidant therapy has been studied for several decades, but its cellular effects are global, and it does not show any clinical significance. Recently, it was reported that in the sugen/hypoxic PAH model, the chemical antioxidant TEMPOL failed to attenuate right ventricular hypertrophy and pulmonary arterial remodeling [14]. A targeted approach in antioxidant therapy is a promising technique and could maximize the potential of an antioxidant. Nitroxides are the family of free radical compounds, which are effective scavengers of ROS [15]. Nitroxides function as a superoxide dismutase (SOD) mimetic, performing the interconversion of an oxammonium cation or hydroxylamine [16]. These properties make nitroxide suitable for the development of defensive peptides for preventing Akt nitration in our study. The affinity peptide that is exclusively targeted to protect the Akt nitration site is conjugated with a nitroxide moiety, forming a nitroxide peptide (NP). Specifically, this would block the tyrosine (Tyr)-350 nitration of Akt, and the conjugated nitroxide antioxidant part would effectively alleviate free radical attacks. In this study, we infer that preventing Akt nitration with antioxidant NPs may be an effective remedy in controlling metabolic reprogramming, anaplerosis, and vascular remodeling events in PAH.

## 2. Materials and Methods 

### 2.1. Development of Nitroxide-Conjugated Affinity Peptide

Nitroxide peptide (NP) was synthesized and purified by the 21st Century Biochemicals company (Marlboro, MA, USA) from two parts, one being the peptide part with the affinity to Akt near the Tyr 350 residue (Ser-Arg-Ile-Arg-Ser), and the other being a conjugated antioxidant, nitroxide (3-carboxy-2,2,5,5-tetramethyl-3-pyrroline-1-yloxy), covalently attached to the free N-terminal amine. The purified peptide was stored at −80 °C as a lyophilized powder. Samples were weighed and freshly prepared just before each experiment or injection.

### 2.2. SIN-1 Induced Akt Nitration Assay

Human pulmonary artery endothelial cells (HPAECs) purchased from Lonza, Greenwood, SC, were cultured using an endothelium media specific for HPAEC with 10% FBS. Cells were used from passages 3–6. All experiments were performed on 80%–90% confluent cells. Akt nitration was induced by treatment with 3-morpholinosyndnomine (SIN-1) (1 mM) for 1 h. NP pretreatment groups were treated with 1 µM NP for 30 min, and then cells were washed in PBS and treated with SIN-1 for 1 h. All treatments were accompanied by including controls with the corresponding vehicle. After treatment, the cells were washed in PBS and lysed in RIPA buffer containing protease and phosphatase inhibitor cocktail [17].

### 2.3. Human Subject

Deidentified lung samples consisted of patients with a diagnosis of group I PAH (idiopathic pulmonary arterial hypertension (IPAH) group, N = 10) and healthy controls (control group, N = 10) and were obtained through Pulmonary Hypertension Breakthrough Initiative (PHBI). The PHBI study protocol was approved by the Institutional Review Boards of the participating sites in the network, and all sites were adherent to the requirements of the U.S. Federal Policy for the Protection of Human Subjects (45 CFR, Part 46), and supported the general ethical principles of the Declaration of Helsinki.

### 2.4. Rat Model of PH

Female Sprague Dawley rats (200–250 g) obtained from Charles River (Wilmington, MA, USA) were used in this study. Animals were housed at 22 °C, 12 h light/dark cycle, and had access to standard rodent food and water ad libitum. All experimental procedures were approved by the University of Arizona Institutional Animal Care and Use Committee. Generally, PAH was induced by a single injection of SU5416 (50 mg/kg) subcutaneously, followed by 3 weeks of hypoxia (10% O2) and 2 weeks of normoxia. This study included five animal groups: control group; SU1, rats were analyzed after 1 week of SU5416 and hypoxia treatment; SU2, rats were analyzed after 2 weeks of SU5416 and hypoxia treatment; SU5, rats were analyzed after 5 weeks of SU5416 treatment (3 weeks of hypoxia with a following 2 weeks of normoxia); and SU2+NP, peptide treatment 0.1 mg/kg/d intravenous (i.v.) injection started with SU5416 treatment for 2 weeks. 

### 2.5. Hemodynamic Measurement 

Animals were anesthetized with Inactin 100 mg/kg intraperitoneally (i.p.) (T133, Sigma-Aldrich, St. Louis, MO, USA). A customized pressure transducer catheter (SPR-513, Millar Instruments, Houston, TX, USA) was inserted into the RV via the right jugular vein and advanced into the right ventricle to monitor right ventricular systolic pressure (RVSP), as described previously [7,18]. Briefly, the pressure transducer catheter was connected to a Millar Transducer Control Unit TC-510 and PL3504 PowerLab 4/35 data acquisition system (AD Instruments, Colorado Springs, CO, USA) to monitor RV pressure for 30 min. After this, a tracheal catheter was connected to a ventilator system (Harvard Rodent Ventilator-683; Harvard Apparatus, South Natick, MA, USA), the thorax was opened, and the lungs flushed with 0.9% sodium chloride through the right ventricle (RV). Heart and lungs were collected from animals; the RV free wall was separated from the left ventricle (LV) and the septum (S). Fulton index (RV/ LV + S ratio) as a parameter of RV hypertrophy was calculated. The total wet lung weight was measured and normalized by the body weight of the animal. The left lung was fixed in formalin and embedded in paraffin for histological examination. The other portion of the lung was stored at −80 °C for further biochemical studies.

### 2.6. Histological Analysis 

For the morphometric assessment of pulmonary vessels, 5 μm tissue sections were dewaxed and stained with Verhoeff–Van Gieson (EVG) elastic fibers stain, by HistoWiz Inc. (Brooklyn, NY, USA), using standard operating procedures and a fully automated workflow system. Ten transversely sectioned pulmonary arteries (diameter < 300 μm) within each category per each animal (*N* = 6 per group) were randomly selected from the whole-slide 40× digitized image Aperio AT2 scanner (Leica Biosystems, IL, USA). Immunohistochemistry was performed using standard protocols. The sections were deparaffinized and incubated with primary antibodies against Akt Y350 NO2 (1:100) and Ki67 (1:800) (ab15580 Abcam) and Dab Rabbit H1 (pH 6) for 20 min. The morphometric analysis was done by an investigator in a blinded grouping fashion. The wall thickness of the pulmonary artery (PA) was measured using the publicly available software Fiji ImageJ (http://fiji.sc/Fiji; in the public domain) [19]. 

### 2.7. Western Blot Analysis

For an analysis of the total lung protein, lung tissues were lysed as previously described [20]. Briefly, 20–40 mg of lung tissue (human/rat) was lysed in permeabilization buffer mixed with protease and phosphatase inhibitor cocktails (78429, Thermo Scientific, Rockford, IL, USA) using a Fisher Homogenizer 850. The homogenate was centrifuged at 10,000× *g* for 10 min, and the supernatant was carefully collected. Cell membrane and cytosolic fractions were isolated using the FractionPREPTM cell fraction kit (Biovision, Milpitas, CA, USA) according to the manufacturer’s instruction. The protein concentration was measured using the BCA protein assay kit (Thermo Scientific, Rockford, IL, USA). Samples were incubated with 6x Laemmli sample buffer (Boston Bioproducts Inc., Ashland, MA, USA) for 5 min at 95 °C, loaded on the 4%–20% SDS-PAGE Mini-PROTEAN TGX Stain-FreeTM gels (Bio-Rad Laboratories Inc., Hercules, CA, USA), and separated by electrophoresis. Protein bands were transferred using the Trans-Blot Turbo transferring system (Bio-Rad Laboratories Inc.) and then blocked with 5% bovine serum albumin (37525, Thermo Scientific, Rockford, IL, USA) in Tris-buffered saline. Membranes were probed using antibodies against Glut4 (07-1404, 1:1000) from Millipore; pyruvate carboxylase (PC) (Ab229267, 1:1000) and peroxisome proliferator-activated receptor gamma coactivator 1-alpha (PGC-1α) (Ab191838, 1:1000) from Abcam; and pyruvate dehydrogenase (PDH) (45-6600, 1:1000) from Invitrogen; phospho Akt (Ser 473) (4060S, 1:2000), Akt (9272S, 1:1000), hexokinase 1 (HK1) (C35C4, 1:1000), glyceraldehyde-3 phosphate dehydrogenase (GAPDH) (2118S, 1:1000), lactate dehydrogenase-A (LDHA) (2012S, 1:1000), phospho glycogen synthase kinase (GSK) 3 β (Ser9) (9336S, 1:1000), GSK3 β (9315S, 1:1000), and glucose 6-phosphate dehydrogenase (G6PD) (8866S, 1:1000) from Cell Signaling Technology. Mouse monoclonal anti-nitrotyrosine antibodies were obtained from Calbiochem, La Jolla, CA, USA (487923, 1:500). Akt Y350 NO2 antibody was obtained as a custom-made antibody against Akt1-nitro (341-353), and the sequence was CGRLPF-nitroY-NQDHEK. Two peptides were developed with and without Y350 nitration. The antibody developed against nitrated peptide was cleaned using non-nitrated peptide (Akt1 (341-353): CGRLPFYNQDHEK) to remove unspecific antibodies. Affinity purification was done using large, reusable affinity purification columns against the unmodified and nitrated peptides. ELISA validation showed specificity towards the nitrated sequence and, therefore, selectively purified antibodies raised to nitro-Akt (Pacific Immunology Corp. Ramona, CA, USA). Since the quantification of Akt nitration was not fully established by mass spectrometry, we used a common method of a newly developed antibody for the quantification of the specific Y350 nitration of Akt. The reactive bands were visualized by the chemiluminescent ChemiDoc^TM^ MP Imaging System (Bio-Rad Laboratories Inc., Hercules, CA, USA) and analyzed using Image Lab^TM^ software. The protein loading was normalized per total sample of protein as a fold control using stain-free gels as previously described [21]. This normalization is equal to housekeeping genes normalization and has been rigorously evaluated by Bio-Rad company (http://www.bio-rad.com/en-us/applications-technologies/stain-free-imaging-technology?ID=NZ0G1815) and by our lab in comparison with beta-actin normalization.

### 2.8. Seahorse Assays

For the glycostress Seahorse assay, human pulmonary artery smooth muscle cells (HPASMCs) (purchased from Lonza, Greenwood, SC, USA) were seeded in a 24-well Seahorse cell culture microplate at 50,000/well and kept overnight to form a monolayer. On the day of experiment, media were aspirated and cells were incubated at 37 °C in a non-CO2 incubator for 1 h with 0.5 mL XF base medium (cat# 102353-100, Agilent, Santa Clara, CA, USA) supplemented with 2 mM glutamine. Glucose (56 µL, 100 mM), oligomycin (62 µL, 100 µM) and 2-deoxyglucose (69 µL, 500 mM) were added to the flux pack wells (cat# 102342-100, Agilent, Santa Clara, CA, USA). ECAR (extracellular acidification rate) was then measured using the Seahorse Bioscience XFe24 extracellular flux analyzer (Agilent, Santa Clara, CA, USA) according to the manufacturer’s protocol. For the mitostress assay, cells were prepared similarly to the glycostress assay, with the exception that the XF base incubation media was supplemented with pyruvate (1 mM), glutamine (2 mM), and glucose (10 mM). Oligomycin (56 µL, 10 µM), FCCP (62 µL, 10 µM) and rotenone + antimycin-A (69 µL, 5 µM) were added to the flux pack wells. OCR (oxygen consumption rate) was then identified according to the manufacturer’s protocol. Data were normalized to cell number as determined by flow cytometry. All the chemicals used for these experiments, unless specified, were procured from Sigma-Aldrich, Saint Louis, MO.

### 2.9. Lactate Dehydrogenase Assay

LDH activity in lung tissue was assessed using the Lactate Dehydrogenase Activity Colorimetric Assay Kit (K726, BioVision Inc. CA, USA) according to the manufacturer’s instruction. All values were normalized with respect to protein concentrations as determined by the Pierce^TM^ BCA Protein Assay Kit (Thermo Scientific, Rockford, IL, USA).

### 2.10. Pyruvate Dehydrogenase Assay

Lung tissue was lysed using RIPA buffer, and the lysate was used for the PDH activity. PDH activity was measured with the PDH Activity Assay Kit (MAK183, Sigma-Aldrich, Saint Louis, MO, USA) per manufacturer protocol. All values were normalized with respect to protein concentrations as determined by the Pierce^TM^ BCA Protein Assay Kit (Thermo Scientific, Rockford, IL, USA).

### 2.11. Metabolic Intermediates Analysis

Data were acquired using the West Coast Metabolomics Center at UC Davis. Briefly, a Restek corporation Rtx-5Sil MS column was used with a helium mobile phase at the temperature interval 50–330 °C and flow-rate 1 mL min^−1^. Injection volume was 0.5 μL at 50 °C ramped to 250 °C by 12 °C s^−1^. Oven temperature started at 50 °C for 1 min, then ramped at 20 °C min^−1^ to 330 °C, and held constant for 5 min. Mass spectrometry analysis was done on a Leco Pegasus IV mass spectrometer with unit mass resolution at 17 spectra s^−1^ from 80 to 500 Da at −70 eV ionization energy and 1800 V detector voltage with a 230 °C transfer line and a 250 °C ion source. Raw data files were preprocessed directly after data acquisition and stored as ChromaTOF-specific *.peg files, as generic *.txt result files, and additionally as generic ANDI MS *.cdf files. ChromaTOF ver. 2.32 was used for data preprocessing without smoothing, 3 s peak width, baseline subtraction just above the noise level, and automatic mass spectral deconvolution and peak detection at signal/noise levels of 5:1 throughout the chromatogram. Apex masses were reported for use in the BinBase algorithm. Resulting *.txt files were exported to a data server with absolute spectra intensities and further processed by a filtering algorithm implemented in the metabolomics BinBase database. Significantly changed metabolites were processed with principal component analysis (PCA).

### 2.12. Statistical Analysis

Statistical analysis was performed using GraphPad Prism version 5.01 (GraphPad Software, San Diego, CA, USA). Outliers were identified with Grubbs’ test using GraphPad outlier calculator (alpha = 0.5) at https://www.graphpad.com/quickcalcs/Grubbs1.cfm. The mean value (± SE) was calculated for all samples, and significance was determined by the unpaired t-test, Mann–Whitney U t-test, or analysis of variance (ANOVA). For one-way ANOVA, Bonferroni multiple comparison tests to compare the selected pairs of columns was used. The significance was calculated using a 95% confidence interval. 

## 3. Results

### 3.1. Inhibition of Akt Nitration by NP

**Figure 1:**
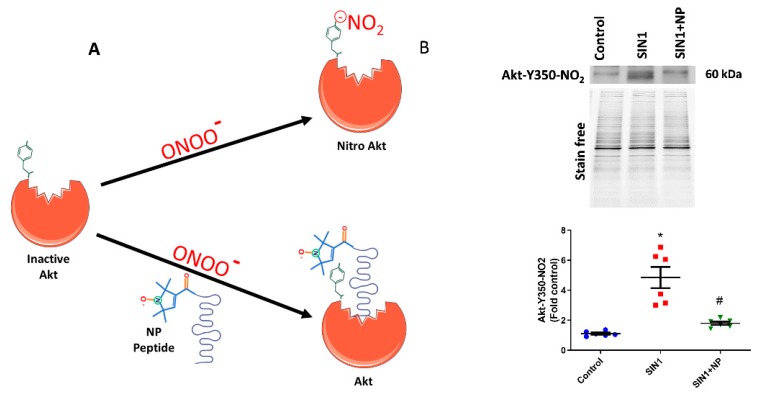
Nitroxide-conjugated peptide blocked nitration of Akt. (**A**) Schematic diagram of nitroxide antioxidant conjugated peptide binding at tyrosine 350 residue of Akt and inhibition of peroxynitrite radical induced Akt nitration. (**B**) SIN-1 (1 mM) for 1 h significantly increased Y350 nitration of Akt in human pulmonary artery endothelial cells (HPAECs), and pretreatment with nitroxide peptide (NP) showed a 55% reduction in Akt nitration. Also, this demonstrates the specificity of our antibody towards Y350-nitroAkt. Data expressed as mean ± SE normalized on total protein (stain free); N = 6; * *p* < 0.05 versus control; # *p* < 0.05 versus SIN-1 by ANOVA.

In the current investigation, we treated the sugen/hypoxia PAH model with our newly designed defensive peptide, which blocks the Akt nitration site at Y350. This peptide has two parts, one being the peptide part that has an affinity to Akt (Ser-Arg-Ile-Arg-Ser or SRIRS), and the other being a conjugated nitroxide antioxidant. Figure 1A depicts the functioning of the defensive peptide; the peptide part specifically binds near to the Tyr-350 residue of Akt and masks it from nitration (Figure 1A). The nitroxide antioxidant part conjugated to the affinity peptide would help scavenge free radicals from tyrosine residue and thereby prevent Akt modifications by peroxynitrite. The efficiency of the developed defensive nitroxide peptide was tested by treating HPAECs. Initially, we treated HPAECs with a peroxynitrite donor, SIN-1, which increased the Akt nitration. The amount of Y350 Akt nitration was recognized by using the custom-made antibody specific towards the nitrated Y350 sequence of Akt, which showed a very clear increase in Akt nitration in SIN-1-treated cells (Figure 1B). NP was incubated with HPAECs for 30 min, then it was washed with PBS, and finally SIN-1 was added to cells. Interestingly, we found that NP treatment showed 55% reduction of Akt nitration in HPAECs (Figure 1B). Also, this additionally demonstrates the specificity of our antibody towards recognition of nitro-Y350 Akt as well as efficacy of NP in blocking the nitration of Akt at the Y350 residue in pulmonary endothelial cells. Utilization of antibody is commonly used for quantification of post-translational modification since mass spectrometry analysis is not a quantitative method, especially for nitration modification of proteins. 

### 3.2. Akt Nitration is an Early Event Initiating PAH

**Figure 2:**
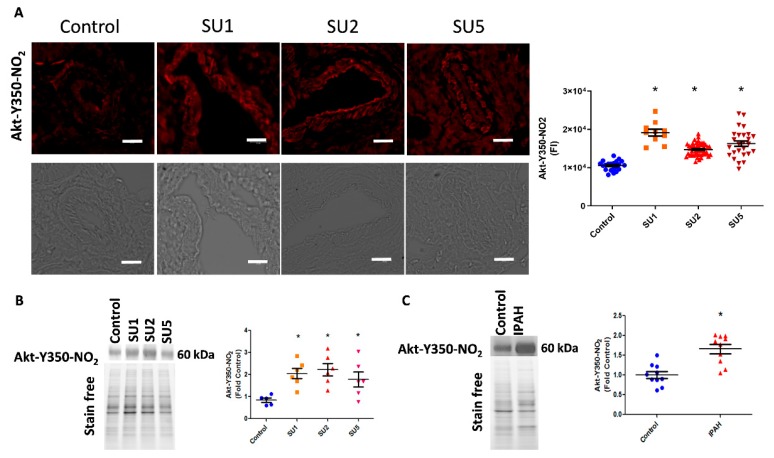
Akt nitration, an early event in PAH. (**A**) Immuno-fluorescence imaging indicated increased Akt nitration in the endothelial and smooth muscle layer of pulmonary arteries of rats after one, two, and five weeks of PAH progression (scale bar is 20 µm). FI, fluorescent intensity; SU1, rats were analyzed after 1 week of SU5416 and hypoxia treatment; SU2, rats were analyzed after 2 weeks of SU5416 and hypoxia treatment; SU5, rats were analyzed after 5 weeks of SU5416 treatment (3 weeks of hypoxia with a following 2 weeks of normoxia). Mean ± SE, *N* = 10–50 arteries, * *p* < 0.05 versus control by ANOVA. (**B**) Western blot analysis showed significantly increased expression of nitroY350 Akt in sugen treatment groups. Data expressed as mean ± SE, *N* = 6–8, * *p* < 0.05 versus control by ANOVA. (**C**) Western blot analysis of healthy controls and idiopathic pulmonary arterial hypertension (IPAH) patients indicated significantly increased nitroY350 Akt signal in IPAH. Data expressed as mean ± SE, *N* = 10, * *p* < 0.05 versus control by Mann–Whitney U t-test.

In the present study, we also screened the onset of Akt nitration in three time phases after sugen injection: early, after one week; two weeks; and finally at five weeks. Interestingly, in immunofluorescence imaging, we found increased nitration of Akt in endothelial and smooth muscle layers of pulmonary arteries. This was observed as early as week one of the disease induction and trails in two and five weeks of the disease (Figure 2A). We also correlated this observation with increased nitro Akt protein modification in lung lysates, in one, two, and five weeks of the sugen PAH model progression (Figure 2B). This observation signifies that the nitration of Akt starts early in the pathogenesis of PAH. Importantly, screening of human lung tissue samples from the PHBI cohort also revealed increased Akt nitration compared with healthy controls (Figure 2C). 

**Figure 3:**
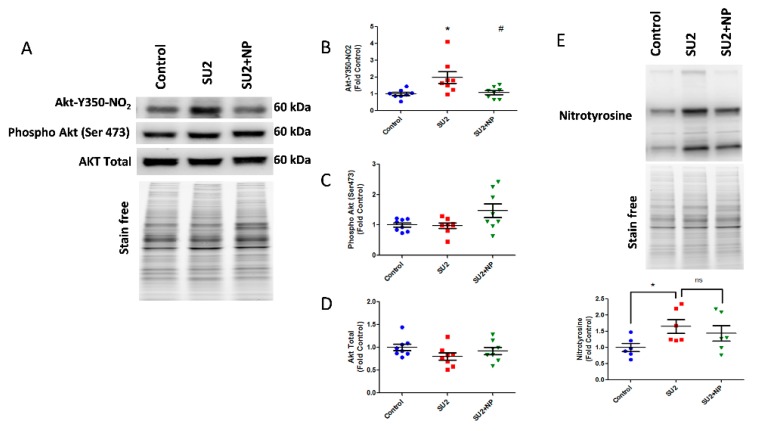
NP treatment attenuated Akt nitration in early PAH. (**A**) Western blot of nitro Akt, phospho Akt, and total Akt expression in the lung lysate of sugen two weeks and sugen two weeks plus NP treatment groups. (**B**) NP treatment significantly reduced nitro Akt modification. Phosphorylation of Akt at Ser-473 (**C**) and total Akt expressions (**D**) were not altered in the two-week sugen/hypoxia model and NP treatment. (**E**) Total nitration in lungs was increased in the SU2 group, and NP treatment did not attenuate the total nitration in the lung significantly; thus, this shows NP selectivity toward Akt nitration. Data expressed as mean ± SE normalized on total proteins, *N* = 6–8, * *p* < 0.05 versus control, # *p* < 0.05 versus SU2 by ANOVA.

Based on these findings, we treated sugen/hypoxia rats with NP 0.1 mg/kg/d i.v. for two weeks. The NP treatment showed a remarkable reduction of nitroAkt modification in the lung tissue (Figure 3A). Here, NP significantly blocked Akt nitration at the inception of PAH development (Figure 3B). Phosphorylation of Akt at Ser-473, an important triggering factor in PAH pathogenesis [5], was found not altered in the two-week sugen/hypoxia model. Also, the NP treatment did not change the total Akt expression (Figure 3C,D). These results suggest that the nitration of Akt is a primary episode in PAH pathogenesis, and masking the nitration of Akt with NP does not affect the phosphorylation levels nor the total expression of Akt. Importantly, total lung nitration levels were increased in the SU2 group, and treatment with NP did not significantly alter total nitration, confirming specificity toward Akt nitration (Figure 3E).

### 3.3. NP Attenuated Ventricular Pressure and Histological Changes

**Figure 4:**
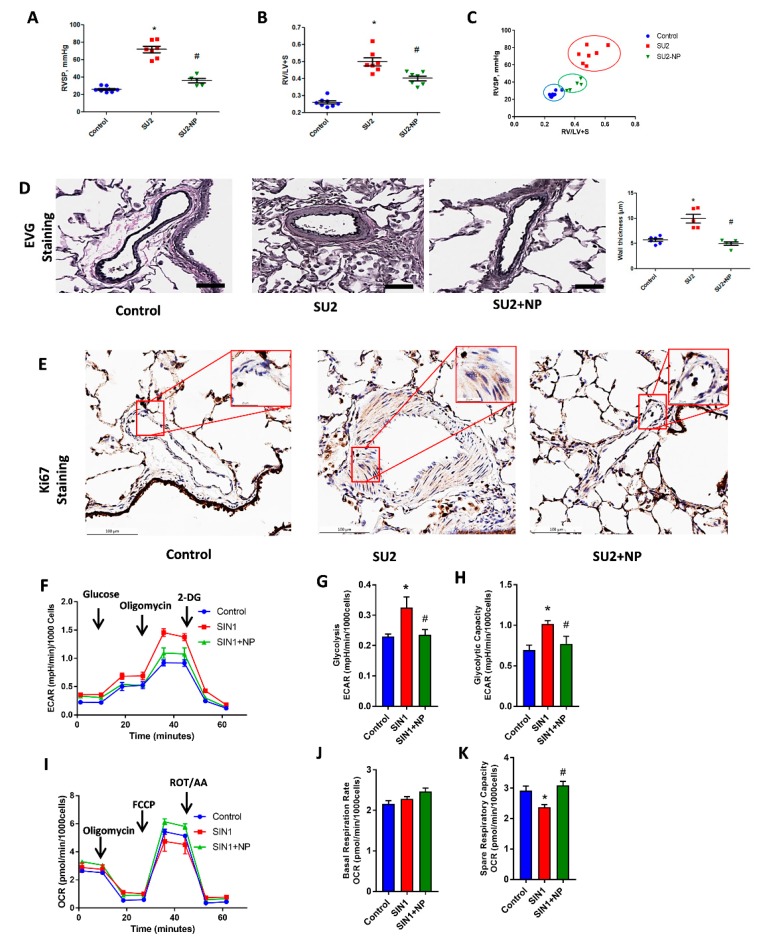
NP controlled hemodynamic and histological alterations. (**A**) Right ventricular systolic pressure (RVSP) was significantly increased in SU2, and NP treatment showed a reduction in RV pressure. (**B**) Fulton index, the ratio of right to left ventricles plus septum (RV/(LV+S)), was significantly increased in SU2 and markedly decreased in NP treatment. (**C**) Correlation analysis between RVSP and Fulton index showed a significant attenuation of the PAH phenotype in NP treatment. (**D**) Clastic Verhoeff–Van Gieson (EVG) staining exhibited perivascular fibrosis, vasoconstriction, and proliferation in pulmonary arteries of SU2, and these alterations were significantly attenuated with NP treatment (scale is 100 µm). (**E**) Ki-67 immunohistochemical staining showed increased cellular proliferation in the media of the pulmonary artery in the SU2 group, and NP effectively prevented vascular remodeling and cellular proliferation (scale is 100 µm). Small box represents magnified images at 25 µm. Data expressed as mean ± SE, *N* = 4–7, * *p* < 0.05 versus control, # *p* < 0.05 versus SU2 by ANOVA. (**F**) Glycolytic rate of HPASMCs; Sin-1 treatment increased glycolysis, but NP treatment attenuated this back to control levels. ECAR, extracellular acidification rate; DG, deoxy glucose. Data expressed as mean ± SE, *N* = 6–7. (**G**) Glycolysis and (**H**) glycolytic capacity increased with SIN-1 treatment and was found to be attenuated with NP treatment. (**I**) Mitochondrial oxidative phosphorylation rate decreased with SIN-1 treatment, and NP treatment upregulated the oxidative phosphorylation rate to control levels in HPASMCs. OCR, oxygen consumption rate; FCCP, trifluoromethoxy carbonylcyanide phenylhydrazone; ROT/AA, rotenone/antimycin-A. Data expressed as mean ± SE, *N* = 6–7. NP treatment without affecting any basal respiration rate (**J**) significantly attenuated the SIN-1-increased spare respiratory capacity (**K**) back to control levels.

RV function in PAH was screened in the two-week sugen/hypoxia with NP treatment model. Hemodynamic measurement by RV catheterization indicated an increase in RVSP in the two-week sugen/hypoxia PAH model. NP treatment showed a reduction in RV pressure from 71.9 ± 3.6 to 36.1 ± 2.6, *p* < 0.001 (Figure 4A). In accordance with increased pressure, the RV of two-week PAH showed an increase in the right heart hypertrophy, evidenced by Fulton index (Figure 4B), and this was found reduced with NP treatment. Figure 4C shows a significant correlation between RVSP and the Fulton index in the SU/hypoxia model, and early NP intervention prevented both remodeling and heart hypertrophy back to almost control levels. 

We studied the histological alterations in lung tissue associated with increased RV pressure in the two-week sugen/hypoxic PAH model. EVG staining showed perivascular fibrosis, vasoconstriction, and proliferation in pulmonary arteries. This signifies vascular obstruction and increased RV pressure overload in the pulmonary artery (Figure 4D). Ki-67 immunohistochemical staining, a crucial protein marker for cellular proliferation, was found increased in smooth muscle cells of the pulmonary artery in the rat PAH model. NP effectively prevented vascular remodeling and cellular proliferation in PAH (Figure 4E). These observations suggest that NP reduced proliferative pathological changes and maintained vascular histology, resulting in reduced RV pressure and thereby preventing PAH development. 

Concurrent to proliferative phenotypical changes in smooth muscle cells of the pulmonary artery, we observed increased glycolysis and decreased oxidative phosphorylation in HPASMCs treated with the peroxynitrite donor SIN-1. SIN-1 treatment enhanced the overall glycolysis and glycolytic capacity in HPASMC, as demonstrated in Figure 4F–H. NP treatment attenuated nitration-induced glycolysis and brought it back to control levels by inhibiting Akt nitration. Moreover, SIN-1 treatment attenuated mitochondrial respiration, but on the other hand, NP treatment prevented SIN-1-induced changes in mitochondrial respiration by significantly enhancing mitochondrial function, as demonstrated by spare respiratory capacity, without affecting the basal respiration rate (Figure 4I–K). These observations explain a concise metabolic transition in smooth muscle cells from oxidative phosphorylation to glycolysis, similar to highly proliferative cancer cells.

### 3.4. NP Treatment Controlled Glucose Uptake and Glycolysis in PAH 

**Figure 5:**
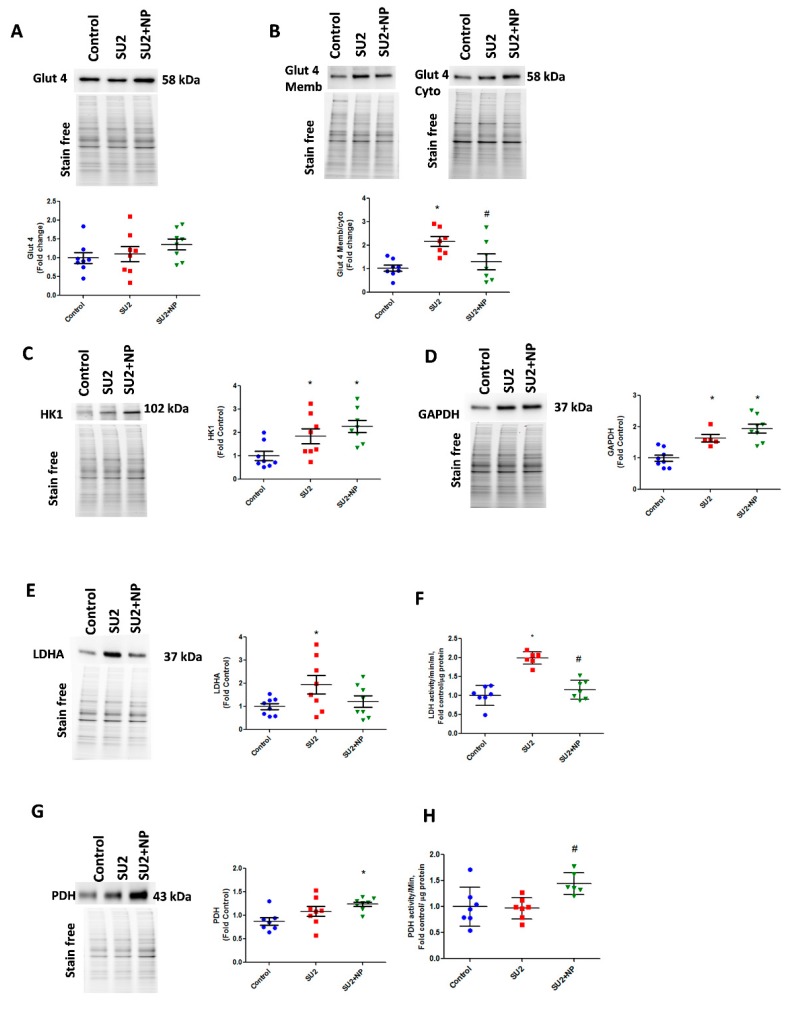
NP treatment balanced glucose influx and glycolysis in PAH. **(A**) Western blot of total glucose transporter Glut4 expression in control, SU2, and NP treatment. (**B**) The ratio of Glut4 expression in membrane/cytosol was found significantly increased in SU2, and NP treatment controlled Glut4 translocation. (**C,D**) HK1 and GAPDH expression increased in sugen/hypoxia treatment represents increased glucose utilization by glycolysis. (**E**) Lactate dehydrogenase expression elevated in SU2 represents increased lactate production in early PAH. (**F**) Increased lactate dehydrogenase activity in lung tissue was attenuated with NP treatment. (**G,H**) Pyruvate dehydrogenase expression and activity were found significantly increased in NP-treated sugen/hypoxia. Data expressed as mean ± SE normalized on the total protein, *N* = 6–8, * *p* < 0.05 versus control, # *p* < 0.05 versus SU2 by ANOVA.

In the early sugen/hypoxic PAH model, we checked glycolytic metabolism, usually found altered in highly proliferative cells. Glucose uptake into the cell is controlled by glucose transporters, and Glut4 is known to be Akt dependent. Akt is an important regulator of Glut4 translocation to the cell membrane. During the first two weeks of the disease, lungs demonstrated the same Glut4 expression, but increased Glut4 localization was identified in membrane fraction. This correlated with the observed increase in Akt nitration, and NP treatment attenuated Glut4 translocation to the membrane (Figure 5A,B). We, therefore, could infer that Glut4 translocation and subsequent increase in glucose uptake was increased due to Akt nitration. The increased glucose concentration in the cytosol and activation of the Akt pathway triggered a high glycolysis rate by upregulating HK1 and GAPDH enzymes (Figure 5C,D). In early PAH, increased glycolysis was found to result in increased production of the aerobic product, lactate from pyruvate, by the enzyme LDHA. SU2 lung tissue showed increased LDHA expression and activity (Figure 5E,F). Prevention of Akt nitration significantly reduced pyruvate disposal to lactate by attenuating LDHA expression and its activity. This resulted in an increased expression of PDH enzyme and its activity to normalize the glycolytic metabolism through oxidative phosphorylation (Figure 5G,H). Downregulation of LDHA decreased acidification rate by lactate, and upregulation of PDH helps to supply increased acetyl Co-A for oxidative phosphorylation in the mitochondria. This explains the Seahorse analysis data in Figure 4F,I. This substantiates that NP treatment potentially balances the glycolytic shift in pulmonary vascular cells of PAH, favoring flux of pyruvate into oxidative phosphorylation and restoring metabolically reprogrammed cells.

### 3.5. Pentose Phosphate Pathway and Glycogenesis Decreased in PAH

**Figure 6:**
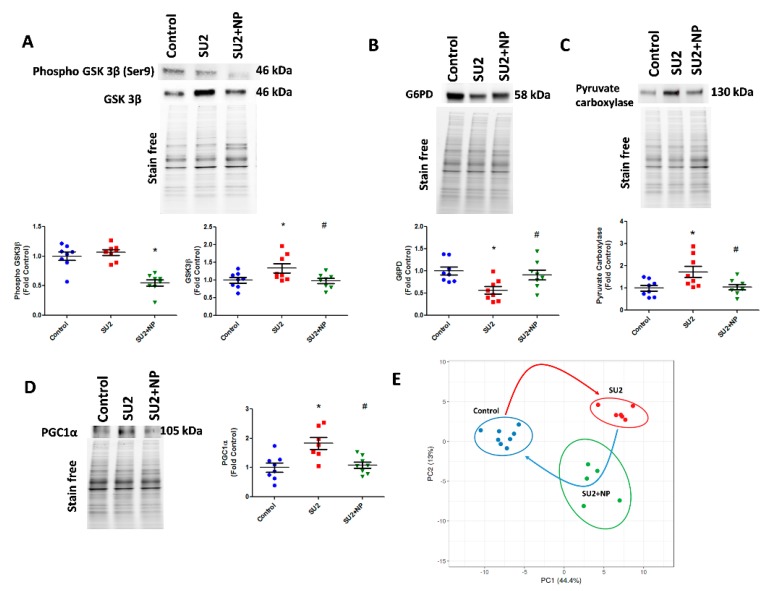
NP maintained the glycolytic shift in early PAH. (**A**) Western blot of phospho GSK3β and GSK3β in SU2 and SU2+NP treatment. Sugen/hypoxia-induced increase in GSK3β expression was reduced with NP treatment. (**B**) Decreased G6PD expression in SU2 was upregulated significantly with NP treatment. (**C**) Increased pyruvate carboxylase expression was decreased with NP treatment. (**D**) Mitochondrial biogenesis marker PGC1α expression found increased in SU2 was significantly reduced with NP treatment. Data expressed as mean ± SE, *N* = 6–8, * *p* < 0.05 versus control, # *p* < 0.05 versus SU2 by ANOVA. (**E**) Metabolite analysis of lung lysate showed metabolic reprogramming in SU2 group and attenuation of metabolic changes by NP treatment. Principal component 1 (PC1) and principal component 2 (PC2) plots explained 44.4% and 13% of the total variance, respectively. Prediction ellipses for control, SU2, and SU2+NP groups show a probability of 0.95 that a new observation from the same group will fall inside the ellipse. *N* = 19 data points.

In accordance with increased glycolysis, we have also screened the compensatory glycogenesis, the anabolic pentose phosphate pathway (PPP), and PC-mediated anaplerosis pathways in the two-week sugen/hypoxia PAH model. GSK3β, which downregulates glycogen synthesis by inhibiting glycogen synthase, was found increased in sugen/hypoxia. Glucose shuttling in PPP from glucose 6-phosphate is driven by G6PD and was found decreased in the two-week PAH model (Figure 6A,B). Interestingly, the inhibition of Akt nitration with NP effectively maintained glycogen synthesis and PPP. The increased glycolytic rate in sugen/hypoxia resulted in the activation of PC, increasing oxaloacetate from pyruvate going to the TCA cycle and, thus, upregulating anaplerosis (Figure 6C). Increased anaplerosis may affect pulmonary vascular cell proliferation in PAH [12]. NP treatment during the first two weeks at 0.1mg/kg/d i.v. resolved anaplerosis by decreasing the expression of PC. Altered oxidative phosphorylation in PAH could result largely in mitochondrial dysfunction. Two weeks of sugen/hypoxia treatment increased PGC-1α expression, a major transcriptional coactivator and a chief compensatory controller of mitochondrial biogenesis in hypoxic stress and cellular proliferation [22]. Importantly, prevention of Akt nitration significantly controlled PGC-1α expression in the lung tissue (Figure 6D). Finally, we undertook lung metabolite profiling using LC-MS quantitative proteomics for control, SU2, and SU+NP groups. Significantly changed (*p* < 0.05) metabolites were analyzed with principal component analysis, and the data showed a significant separation between all groups. In Figure 6E, Control and SU2 groups showed further separation by PCA. NP treatment resulted in moving diseased animals toward the control and can indicate resolution of reprogramed metabolism in the PAH model. Indeed, 30 metabolites were significantly restored by NP treatment. 

## 4. Discussion

**Figure 7:**
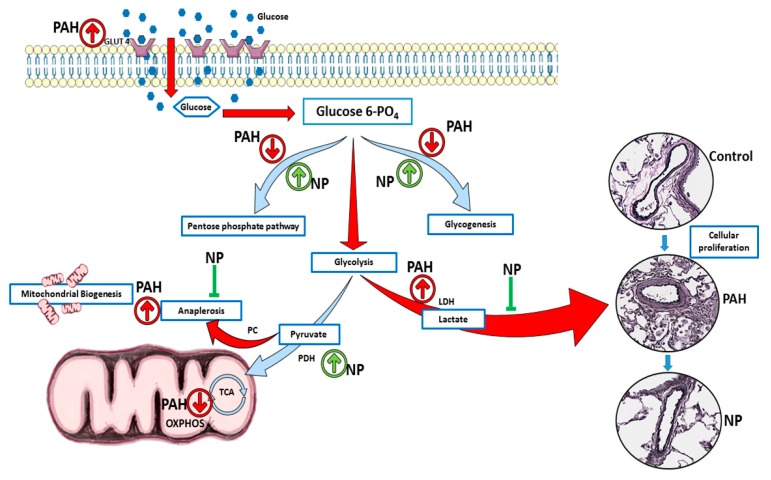
NP attenuated metabolic reprogramming in pulmonary hypertension. In two weeks of sugen/hypoxia, increased nitration induced activation of Akt upregulated the glucose transporter Glut4 to the membrane, and this augmented cellular glucose influx. This results in increased aerobic glycolysis and anaplerosis as well as a reduction in glycogenesis and pentose phosphate pathway. These metabolic derangements result in increased cellular proliferation and vascular remodeling in lung tissue of early PAH. NP treatment balanced cellular glucose influx, glycogen synthesis, and the pentose phosphate pathway. NP corrected the glycolytic shift, improved oxidative phosphorylation, and inhibited anaplerotic reprogramming. Thus, proliferative pathological changes in PAH were found to be prevented with NP treatment. PAH, pulmonary arterial hypertension; NP, nitroxide-conjugated peptide; TCA, tricarboxylic acid cycle; OXPHOS, oxidative phosphorylation.

Pulmonary arterial hypertension (PAH) is a progressive and fatal condition characterized by narrowing of the pulmonary arteries because of muscular thickening and intimal proliferation [23]. Abnormal Akt activation causes normal cells to undergo proliferative conversion [24], but in PAH, the cause and activation of Akt are not fully elucidated. The nitration of Akt plays a crucial role in its activation, and we found increased Akt nitration in patients with PAH as well as in the sugen/hypoxia model. Akt nitration can activate eNOS phosphorylation, and this, in turn, increases peroxynitrite formation, nitrosative stress, and mitochondrial dysfunction. Prolonged mitochondrial dysfunction is responsible for PAH development with vascular damage and metabolic disorder [6,7]. The study of metabolic alterations can be performed either by metabolomics or proteomics analyses. In our present study, we followed protein expression and compared metabolite profiles between groups. This approach clearly demonstrated PAH-specific alterations in the metabolic enzymes and metabolites, unveiling the protective mechanism of the antioxidant peptide in the context of Akt nitration. Interestingly, during the two-week sugen/hypoxic model, we identified that activation of Akt upregulated the glucose transporter Glut4 to the membrane, and this augmented cellular glucose influx. This resulted in increased aerobic glycolysis and anaplerosis, and a reduction in glycogenesis and PPP, similar to highly proliferative cancer cells. Histological studies identified cellular proliferation and vascular remodeling in lung tissue of early PAH. These pathological modifications recapitulate increased RV systolic pressure and Fulton index in early PAH. NP inhibited Akt nitration at Tyr-350 and controlled metabolic derangement in early PAH. NP treatment balanced cellular glucose influx, glycogen synthesis, and PPP. These events controlled glycolysis, oxidative phosphorylation, and inhibited anaplerotic reprogramming. Thus, with NP treatment, proliferative pathological changes in PAH were found to be prevented, and RV functioning improved with normal RVSP. The summary of observed metabolic alterations and effects of NP treatment is combined in Figure 7.

As the incidence of PAH is high in females, we used female rats to study the anaplerotic reprogramming and Akt nitration events in PAH. The high incidence of PAH in females could perhaps be due to increased nitric oxide production and nitrosative stress development [25]. NO, and the superoxide anion produced in hypoxic stress, potentiates a variety of biological processes including peroxynitrite formation, nitration of many tyrosine residues of proteins, and a decrease in antioxidant status [26]. Recently, we unveiled that Akt1 nitration at the Tyr-350 residue enhanced the phosphorylation of eNOS at Ser617/1179 and its translocation to mitochondria in pulmonary artery endothelial cells [6]. Excitingly, in our present investigation, using the newly developed nitro Akt (Y350-NO2) antibody, we discovered that nitration of Akt at Tyr-350 residue was an early molecular modification possibly responsible for the pathogenesis and progression of PAH. Immunohistochemistry and protein expression studies authenticated that Akt nitration started early, from one week of the disease, and trailed up in two and five weeks of the sugen/hypoxic model of PAH. Increased oxidative and nitrosative stress in early sugen/hypoxic PAH could have potentiated these nitration events. Pathological instigation of the Akt pathway has been described in many cancers, specifically in lung carcinogenesis. Importantly, nitrosative stress-induced tyrosine nitration potentiated cellular proliferation, and metastasis [27,28]. 

Identification of early Akt nitration encouraged the design of a targeted NP for its prevention and to study the implication of Akt nitration in PAH pathogenesis. We developed an antioxidant NP having two moieties: one a peptide part, designed complimentary to the Tyr-350 residue of Akt to obtain affinity, and the second, an antioxidant nitroxide that helps to scavenge free radicals/electrons. Nitroxide is a stable free radical, containing a nitroxyl group and an unpaired electron. This can easily diffuse through the cell membranes. In biological settings, they demonstrate SOD and catalase-like activities, preventing the Fenton and Haber–Weiss reactions and mitigating protein and lipid damage [29]. The efficacy of the antioxidant peptide (NP) was screened in HPAECs by inducing nitrosative stress with the peroxynitrite donor SIN-1. Extracellular treatment with NP alleviated (55%) nitration of Akt in endothelial cells. The dose of NP used in this study was 100 times lower than the concentration of the antioxidant, TEMPOL, normally used in treating PAH, and it did not affect the redox homeostasis of the cellular environment. It was also recently reported that TEMPOL treatment was not effective in preventing pulmonary arterial remodeling and, in fact, increased the severity of PAH [14]. 

In accordance with our initial observation, we found that Akt nitration was an early event, and NP was tested in the sugen/hypoxic PAH model at week two of disease progression. Interestingly, we found that NP was effective in blocking the nitration of Akt in early PAH. In late PAH, activation of Akt at Ser-473 plays a crucial role in the reprogramming of metabolic pathways and PAH pathogenesis [5]. Intriguingly, in early PAH, there was neither any phosphorylation at Ser-473 of Akt nor any change in the expression of total Akt. This observation strengthens the idea that the activation of Akt occurring in early PAH might be through nitration of Akt. Akt activation is strongly associated with cellular proliferation and vascular remodeling [30]. Our results suggest that the inhibition of Akt nitration with NP could mitigate early Akt activation and vascular modification. 

Pulmonary vascular remodeling is among the major factors in initiating higher pulmonary vascular resistance and pulmonary arterial pressure. Akt plays a crucial role in hypoxia-induced pulmonary vascular remodeling [5]. In our early sugen/hypoxic PAH models, we found increased RV systolic pressure and right ventricular hypertrophy (Fulton index). RV hypertrophy instigated by increased vascular resistance is primarily a compensatory mechanism, but this often leads to RV dysfunction [31]. In accordance with the increased pressure, the lung tissue of high-pressure rats showed perivascular fibrosis and vasoconstriction in pulmonary arteries. Ki-67, a sensitive protein marker strongly associated with cell proliferation [32], was found to be highly expressed in the pulmonary vasculature. This proliferation in the vascular wall and vasoconstriction explains the reason for elevated RV pressure in early PAH. Fascinatingly, NP treatment improved RV function and pulmonary vascular remodeling in the two-week sugen/hypoxic model. Similarly, in a previous report, inhibition of Akt inhibited muscularization of pulmonary arterioles and reduced right ventricular hypertrophy [33]. These findings suggest the idea that inhibition of Akt nitration with NP could regulate its activation and could prevent downstream cellular proliferative and structural changes in RV and lung vasculature. Moreover, unlike the small molecule inhibitors of Akt, NP can diminish pathological events of nitration-mediated Akt activation without affecting normal Akt function. Thus, we consider this to be an important benefit of the targeted antioxidant approach. 

Previous reports identified that glycolytic shift (Warburg effect) is a major pathogenic cause, in extreme cases, of PAH [10]. Figure 4F,G showed increased glycolytic and decreased oxidative phosphorylation rates in HPASMCs with SIN-1 treatment. This explains Akt nitration induced metabolic reprogramming from oxidative phosphorylation to glycolysis. NP treatment inhibited Akt nitration and attenuated the metabolic reprogramming. Akt activation by peroxynitrite can enhance cellular glucose uptake through the glucose transporter, Glut4, and can increase glycolysis in PAH [34]. In the present study, we profiled Glut4 expression in different cellular fractions and found it was increased in the membrane/cytosolic fraction. In the early two weeks of PAH, nitrated Akt could perhaps aggravate glucose uptake through increased membrane Glut4 translocation. High levels of intracellular glucose influx could upregulate glycolytic metabolism to meet the demands of increased cellular proliferation. This signifies the Seahorse analysis that PAH shifts glycolysis and mitochondrial respiration in the sugen model. Proliferating cells tend to have an increased expression of glucose transporters and glycolytic enzymes to compensate for the increased metabolic needs [35]. Inhibition of Akt nitration with NP maintained glucose influx by downregulating the translocation of Glut4 to the membrane fraction. 

In the present study, glycolytic enzymes HK-1 and GAPDH showed increased expression in lung tissue of the two-week sugen/hypoxia model. HK1 is the first rate-limiting enzyme of glycolysis, and its overexpression can initiate downstream pathways, amplifying reactions that offer increased ATP to proliferating cells [36]. Also, Akt activation can exacerbate glycolysis by GAPDH phosphorylation and activation in hypoxic stress conditions [37]. Here, we speculate that Akt activation by nitration played a cardinal role in cellular glucose uptake and increased glycolysis in proliferating cells of early PAH. LDHA converts pyruvate to more lactate in rapid flux metabolism through glycolysis. Increased LDH expression and activity is characteristic of fast-growing cells, and its suppression stimulates mitochondrial respiration and decreases proliferation [38]. Similarly, in our study, nitration of Akt intersects with the aerobic glycolysis pathway in the two-week hypoxic/sugen model with increased LDHA expression. Increased lactic acid production may result in lactic acidosis, a serious physiological event in PAH [39]. Pyruvate generated in the latter phases of glycolysis is converted to either lactate, via aerobic glycolytic metabolism, or acetyl-CoA, through the oxidation of glucose by PDH. PDH is often considered as the “mitochondrial gate-keeping enzyme”, promoting pyruvate entry into the OXPHOS [40,41]. Interestingly, inhibition of Akt activation with NP increases mitochondrial enzyme PDH, signifying upregulation of oxidative phosphorylation, and a reduction of lactate accumulation. Thus, in our early PAH model, NP treatment efficiently prevented the metabolic reprogramming from glycolysis to oxidative phosphorylation and prevented cellular proliferative changes.

GSK3β is a serine-threonine kinase, regulating a variety of cellular functions like cell division, proliferation, and differentiation. Akt is one of the critical regulators of GSK-3, and phosphorylation and inactivation of GSK-3 represent the anti-apoptotic effects of Akt [42]. In the PAH model, Akt nitration did not upregulate phosphorylation of Ser9 in GSK3β, but the total expression of GSK3β was increased. GSK3β phosphorylation is controlled by phosphorylated Akt, but in early PAH, Akt phosphorylation was found not activated, and this could perhaps be the reason for no change in phosphor-GSK3β expression. Elevation of GSK3β inversely correlates with glycogen synthase [43]. Inhibition of glycogen synthase downregulates glycogen synthesis and could produce more LDH as well as oxaloacetate for aerobic respiration and anaplerosis. However, NP by preventing Akt activation decreased GSK3β expression and maintained glycogen synthesis. On the other hand, we found that G6PD, the rate-limiting enzyme of PPP [44], was decreased. This could downregulate PPP and redirect the metabolism towards increased glycolysis, similar to what we found in a recent complex III-deficient model [7]. In accordance with this, our recent report in PAH patients suggests that G6PD deficiency potentiates hemolysis and disease progression [45]. In the two-week sugen/hypoxic model, nitro Akt activation maintained G6PD expression and corrected the PPP, thus balancing glycolytic metabolism to normal.

Downregulation of glycogen synthesis and PPP in early PAH lungs shifted the metabolism towards glycolysis and perhaps produced increased pyruvate. As observed in our study, pyruvate could have activated PC and produced increased oxaloacetate for the TCA cycle. This could result in increased anaplerosis. Anaplerosis by PC and glutaminolysis are the reasons for pulmonary vascular constriction and increased proliferation in PAH [13]. Increased glycolysis and anaplerosis provides supplementary biomass for cellular proliferation in early PAH. Increased anaplerosis could lead to a reduction in oxidative phosphorylation and cause mitochondrial dysfunction [11]. Two weeks of sugen/hypoxia increased PGC-1α expression in lung tissue, representing impairment in mitochondrial function and mitochondria biogenesis demand. PGC-1α might be increased for compensating the metabolic reprogramming and mitochondrial dysfunction in early hypoxic stress [46]. Activation of Akt is associated with the regulation of PGC-1α [47]. In our early PAH model, activation of Akt by Tyr-350 nitration may have upregulated PGC-1α expression. Excitingly, the prevention of Akt nitration with NP downregulated PGC-1α expression, representing improved mitochondrial functioning and a reduction in cellular anaplerosis. 

## 5. Conclusions

Altogether, our results suggest that inhibition of Akt nitration with targeted NP can prevent metabolic reprogramming and pathophysiological events in early PAH. Specifically, NP prevented the activation of Akt by nitration, which controlled a series of cascades, including the balancing of intracellular glucose influx, lactic acidosis, and anaplerosis. Improved glycogenesis, PPP, and oxidative phosphorylation maintained the cellular metabolic balance and decreased proliferation. Suppressing cellular proliferation reduced RV hypertrophy and pulmonary vascular remodeling. These dramatic events controlled vascular flow and regulated RV pressure back to normal.
